# Safety of traditional Chinese medicine injection based on spontaneous reporting system from 2014 to 2019 in Hubei Province, China

**DOI:** 10.1038/s41598-021-88339-9

**Published:** 2021-04-23

**Authors:** Rui Huang, Yuanxuan Cai, Linhui Yang, Xiaofang Shangguan, Bishwajit Ghose, Shangfeng Tang

**Affiliations:** 1 Tongji Medical College, School of Pharmacy, Huazhong University of Science and Technology, Wuhan, 430030 Hubei People’s Republic of China; 2 Tongji Medical College, School of Medicine and Health Management, Huazhong University of Science and Technology, Wuhan, 430030 Hubei People’s Republic of China

**Keywords:** Drug regulation, Drug safety

## Abstract

Traditional Chinese medicine (TCM) injection is widely used in clinical settings, but its adverse drug reactions (ADRs) can be a serious public health concern. The objective is to study the safety of TCM injection and provide suggestions for clinical use. ADR reports collected by the Hubei Adverse Drug Reaction Monitoring Center from 2014 to 2019 were analysed. The safety of TCM injections was described by descriptive analysis and three signal mining methods, including the reporting odd ratio (ROR), proportional reporting ratio (PRR) and comprehensive standard method (MHRA). The findings indicate that the age groups of 0–10 and 41–80 years had the highest rates of reporting ADRs. A total of 96.41% of the ADRs occurred within one week, mostly on the same day that the injection was administered. Among the 60 TCM injections, Shenmai, Xiangdan, Salvia, Shengmai, Astragalus and Xuebijing injection had an above average ratio of severe ADRs (12.63%). A total of 99.24% of the cases improved after treatment. There were 9 deaths whose ADRs were mainly anaphylactic shock, dyspnoea and anaphylactoid reaction. In signal mining, the three methods produced 19 signals that were the same, and 14 of them were off-label ADRs. The frequency of TCM injections in children and elderly patients should be reduced and monitored strictly. Close observation is necessary during the first seven days after receiving the injection. The clinical use of Shenmai, Xiangdan, Salvia, Shengmai, Astragalus and Xuebijing injections should be investigated. Signal mining and more research are needed on TCM injections.

## Introduction

Modern Chinese medicines include almost all modern preparation forms, such as capsules, inhalants, dropping pills, injections and injectable powder^1^. Among them, injections and injectable powders raise challenges both for pharmacovigilance and drug regulation. Traditional Chinese medicine (TCM) injections, the extension and development of TCM, have a history of more than 70 years of use. Between 2004 and 2007, total sales of TCM injections exceeded $2 billion annually^2^. In 2017, China approved the sale of 134 kinds of TCM injections from 224 manufacturers^3^. Only 5 TCM injections are included in Pharmacopoeia of The People’s Republic of China (2015), and 10 are included in the National Essential Drugs List (2018).

With the development and widespread use of TCM injections, adverse drug reactions (ADRs) have gradually become a public concern. In China, an ADR is defined as the harmful reaction of qualified drugs under normal usage and dosage, which has nothing to do with the purpose of drug use^4^. From 2001 to 2016, the National Medical Products Administration (NMPA) issued 15 notifications of ADRs related to TCM injections, involving 20 types^5^. In 2003, Yuxingcao injection led to 12 cases of anaphylactic shock and 40 cases of dyspnoea, and the use of Yuxingcao injection was finally suspended^6^. On April 20, 2009, National Adverse Drug Reaction Monitoring Center issued a warning that Qingkailing injection may have serious ADRs, as more than one-quarter of the patients who died from the injection were children under 14 years of age^7^. The latest ADR notification related to TCM injection was reported in 2013, showing that there were a total of 3306 cases of safflower injection in the ADR monitoring database in 2012, including 154 severe cases. Recent studies found that ADR reports of TCM injections accounted for more than 50% of the ADR reports of TCMs^3^. Compared with conventional injections, the proportion of severe ADRs in TCM injections was slightly lower (6.02% vs 6.72%), and the proportion of unknown (new) ADRs was much higher (46.74% vs 24.13%)^8^.

ADR of TCM injection is a serious public health problem, and there is no strong evidence for their safety, which is mostly based on long-term clinical practice. Special populations, including new-borns, infants, children, the elderly, pregnant women and lactating women, are restricted in the use of TCM injections, and some of them are forbidden. Therefore, it is necessary to evaluate the safety of TCM injections and encourage their re-evaluation. The purpose of this study was to analyse a database of provincial spontaneous reporting systems (SRSs) and study the safety of TCM injections from all aspects of ADRs.

## Methods

### Data source and preprocessing

The data of the adverse drug reaction reports collected by Adverse Drug Reaction Monitoring Center of Hubei Province from January 2014 to December 2019 were classified and analysed, and spontaneously reported by medical institutions, enterprises, and the public in Hubei.

The data were cleaned and preprocessed to ensure that they were clean and complete. The ADR database includes all reported adverse reaction reports. Reports of TCM injections with the registered category of Chinese medicine and the drug approval number containing “z” in the NMPA were selected for inclusion. Injections with herbal ingredients but registered under the category of chemicals were excluded. The analysis only included reports with certain, probable, and possible relationships of drugs and ADR evaluated by the reporting unit, and excluded reports that were unlikely or impossible to evaluate. Since there was no unified standard for the entry of drug names and ADRs in the report, the drug names registered in the NMPA were used as the standard to unify the generic names and the ADRs and clinical manifestations were organized according to the World Health Organization Adverse Reaction Terms (WHO-ART).

For the death cases, relevant information was detailed and carefully analysed to find other key points that had contributed.

From January 2014 to December 2019, the ADR Monitoring Center collected a total of 420,114 reports. There were 25,416 reports meeting the inclusion criteria. Since there might be two or more ADRs in a report or case, and the occurrence of an ADR in the use of a certain drug was considered an event, 33,446 events were included in the statistics.

### Data analysis

A descriptive analysis of age, sex and occurrence, severity, types and results of ADRs in the reports was carried out.

The amount of each ADR of each TCM injection was sorted for ADR signal mining, which quantifies the qualitative nature of the relationship between drugs and ADRs^9^. In ADR signal mining, the reporting odds ratio (ROR), proportional reporting ratio (PRR) and comprehensive standard method (MHRA) as measures of disproportionality were adopted, which is generally used in this area to detect the imbalance of target events compared with other events in the database^9,10^. When the frequency of the target drug event combination (DEC) is significantly higher and reaches the threshold compared to the background frequency, a signal is considered to be generated^11^. The strength of the association between drugs and ADRs was expressed as the ROR and PRR with 95% confidence intervals (CIs). The fourfold table used in the measures of disproportionality is shown in Table 1. The calculation formulas and the threshold for generating a signal with these three methods are presented in Table 2. In this study, signal mining of a single drug and a single ADR was conducted without considering the combination of drug use and drug interaction.

**Table 1:** The fourfold table used in measures of disproportionality.

Category of drugs	Target ADR N	Other ADRs N	Sum
Target drug	a	b	a + b
Other drugs	c	d	c + d
Sum	a + c	b + d	N = a + b + c + d

**Table 2:** Formulas and criteria for generating signals of ROR, PRR and MHRA.

Method	Formula	Criteria and threshold
ROR	$$\begin{aligned} & ROR = \frac{{\left( {a/c} \right)}}{b/d} = \frac{ad}{{bc}} \\ & SE(\ln ROR) = \sqrt {\left( {\frac{1}{{\text{a}}} + \frac{1}{{\text{b}}} + \frac{1}{{\text{c}}} + \frac{1}{{\text{d}}}} \right)} \\ & 95\% \;CI = e^{{\ln \;ROR \pm 1.96\sqrt {\left( {\frac{1}{{\text{a}}} + \frac{1}{{\text{b}}} + \frac{1}{{\text{c}}} + \frac{1}{{\text{d}}}} \right)} }} \\ \end{aligned}$$	a ≥ 3 and lower limit of 95%CI > 1
PRR	$$\begin{aligned} & PRR = \frac{a/(a + b)}{{c/(c + d)}} \\ & SE(\ln PRR) = \sqrt {\left( {\frac{1}{{\text{a}}} - \frac{1}{{{\text{a}} + {\text{b}}}} + \frac{1}{{\text{c}}} - \frac{1}{{{\text{c}} + {\text{d}}}}} \right)} \\ & 95\% \;CI = e^{{\ln \;PRR \pm 1.96\sqrt {\left( {\frac{1}{{\text{a}}} - \frac{1}{{{\text{a}} + {\text{b}}}} + \frac{1}{{\text{c}}} - \frac{1}{{{\text{c}} + {\text{d}}}}} \right)} }} \\ \end{aligned}$$	a ≥ 3 and lower limit of 95%CI > 1
MHRA	$$\begin{aligned} & PRR = \frac{a/(a + b)}{{c/(c + d)}} \\ & \chi^{2} = \frac{{n\left( {\left| {{\text{ad}} - {\text{bc}}} \right| - \frac{n}{2}} \right)^{2} }}{{\left( {a + b} \right)\left( {a + c} \right)\left( {b + c} \right)\left( {c + d} \right)}} \\ \end{aligned}$$	a ≥ 3, PRR ≥ 2 and χ^2^ ≥ 4

## Result

### Sex and age distributions of ADRs

**Figure 1:**
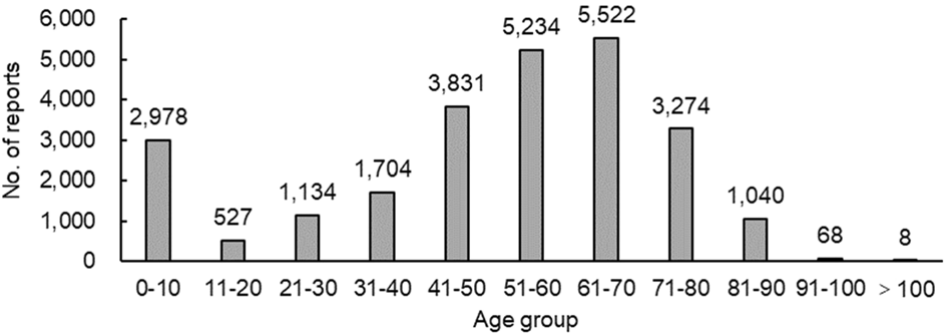
Number of reports in each age group.

Among the 25,416 reports related to TCM injections, except for 46 cases in which the sex was unknown, the number of women (13,632) who had ADRs was slightly greater than that of men (11,738), and the male–female ratio was 1:1.16 with a small discrepancy. The sex differences in specific age groups were more significant. Excluding 50 reports of unknown information, the age groups with higher reporting rates were concentrated in 0–10-year-olds (2978) and 41–80-year-olds (17,861) (see Fig. 1). That is, children and middle-aged and elderly patients were the most common.

### The occurrence of ADRs

**Figure 2:**
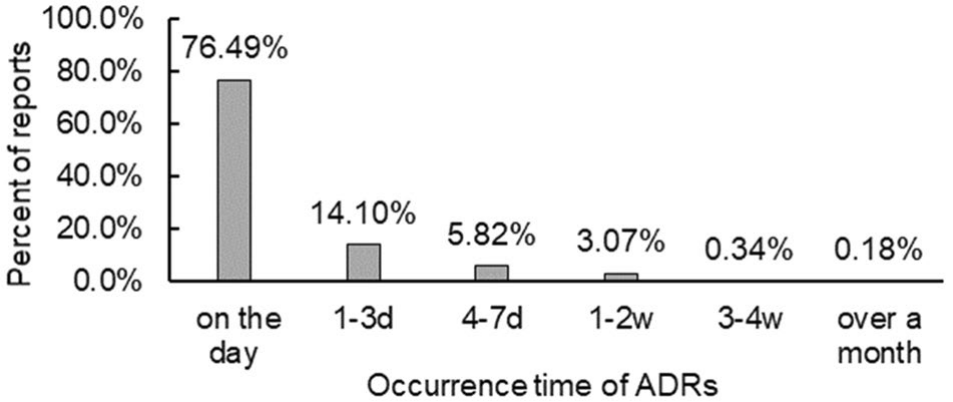
The proportion of the number of reports based on the occurrence time of ADRs (n = 24,707).

The occurrence of ADRs during medication was counted, and reports with obvious errors of entry and whose ADRs occurred after drug withdrawal were excluded. A total of 24,707 reports were included. Figure 2 shows the occurrence time of ADRs after starting the medication. The majority of ADRs occurred on the day of injection (18,898, 76.49%).

### Severity of the reported ADRs

The Administrative Measures on Reporting and Monitoring of ADRs states that according to the severity of ADRs, ADRs were divided into serious and non-serious ADRs. Serious ADRs result in death, life-threatening effects, cancer, a congenital anomaly, birth defects, significant or permanent human disability, damage to organ function, hospitalization or prolonged hospitalization or events that require intervention and treatment to avoid the above results. New and known ADRs are also subdivided according to whether the ADRs are recorded in the drug insert. In addition, ADRs whose types are known but whose severity is greater than that described in the drug insert are also regarded as new ADRs^4^. Serious and new ADRs have always been the focus of ADR research, as they pose a greater threat to the life and health of patients.

**Figure 3:**
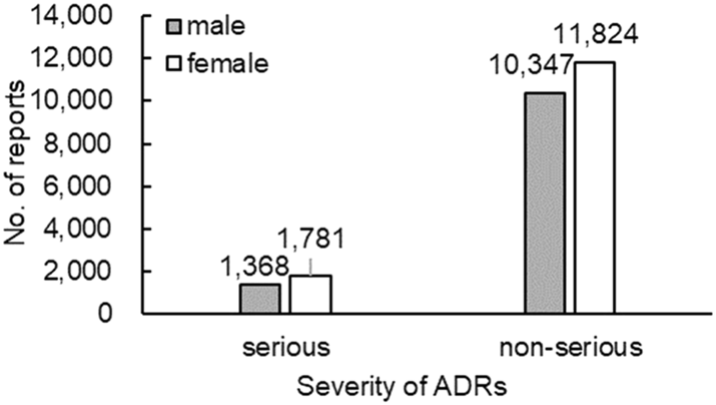
Number of serious and non-serious reports by sex.

**Figure 4:**
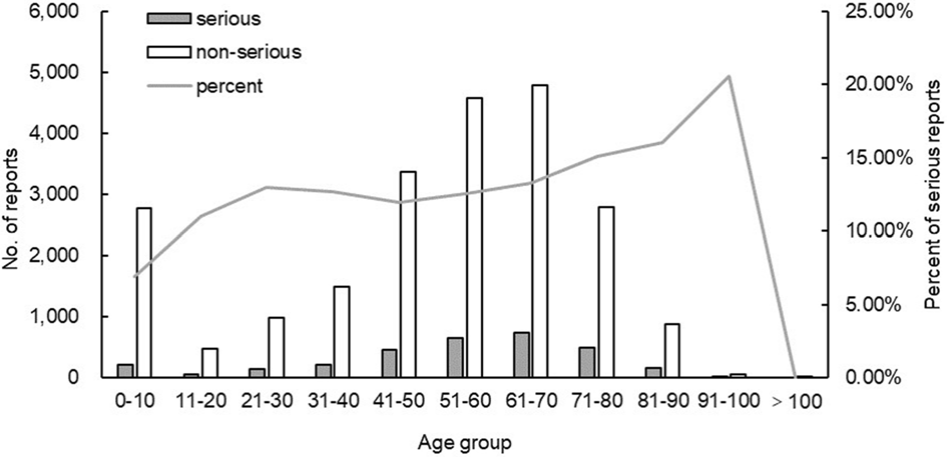
Number of reports and the proportion of serious reports in each age group.

Among the 25,416 reports related to TCM injections, there were 3211 reports of serious ADRs, accounting for 12.63% of the total reports, of which 1223 were new and serious reports. There were 22,205 non-serious reports containing 8244 new and non-serious reports, accounting for 87.37% of the total. The severity of ADRs in males and females is presented in Fig. 3. The severity of ADRs was not significantly different by sex. Figure 4 describes serious and non-serious reports in different age groups and the proportion of serious reports in each age group. In the study, few patients were over 100 years old (8 cases), all of whom had non-serious ADRs. Regardless of patients older than 100 years, it is worth noting that the proportion of serious reports steadily increased with age.

Table 3 shows the number and proportion of serious and non-serious reports by TCM injections (top 15 ordered by the number of serious reports). A total of 60 TCM injections were involved in the study. Among them, Shenmai injection, Xiangdan injection and Qingkailing injection had the most severe reports. It is worth mentioning that the proportions of severe reports of Shenmai injection, Xiangdan injection, Danshen injection, Shengmai injection, Huangqi injection, and Xuebijing injection were higher than the overall proportions (12.63%).

**Table 3:** Number and proportion of serious and non-serious reports by TCM injections (top 15).

TCM injections	Serious N (%)	Non-serious N (%)	Total
Shenmai injection	541 (15.45)	2961 (84.55)	3502
Xiangdan injection	465 (16.11)	2422 (83.89)	2887
Qingkailing injection	368 (12.62)	2547 (87.38)	2915
Panax notoginseng saponins injection	247 (10.86)	2028 (89.14)	2275
Salvia injection	212 (15.28)	1175 (84.72)	1387
Tanreqing injection	176 (10.33)	1528 (89.67)	1704
Safflower injection	142 (11.14)	1133 (88.86)	1275
Xueshuantong injection	129 (9.56)	1220 (90.44)	1349
Xiyanping injection	109 (8.27)	1209 (91.73)	1318
Shengmai injection	108 (15.02)	611 (84.98)	719
Astragalus injection	93 (18.53)	409 (81.47)	502
Reduning injection	86 (11.03)	694 (88.97)	780
Dan hong injection	62 (11.36)	484 (88.64)	546
Shuanghuanglian injection	54 (9.54)	512 (90.46)	566
Xuebijing injection	44 (18.49)	194 (81.51)	238

### Frequently reported ADRs

A total of 33,446 events involved a total of 28 system-organ damage reports, mainly including skin and appendage damage, body as a whole-general disorders and autonomic nervous system disorders. The detailed number and proportion of events are shown in Table 4.

**Table 4:** Number and percentage of ADRs related to system-organ damage (top 10, n = 33,446).

Rank	System-organ damage	N	Percentage
1	Skin and appendage disorders	12,181	36.42
2	Body as a whole-general disorders	8197	24.51
3	Autonomic nervous system disorders	4167	12.46
4	Gastrointestinal system disorders	3221	9.63
5	Central and peripheral nervous system disorders	2526	7.55
6	Respiratory system disorders	2131	6.37
7	Vascular (extracardiac) disorders	222	0.66
8	Psychiatric disorders	202	0.60
9	Application site disorders	136	0.41
10	Metabolic and nutritional disorders	127	0.38

According to the statistics, a total of 209 ADRs were identified, which were concentrated in skin reactions such as rash and pruritus, anaphylactoid reaction of systemic damage and palpitation related to heart rate and arrhythmia. Table 5 shows the distribution of the number of ADRs in the top 95%. Anaphylactoid reaction and anaphylactic shock were the most concerning ADRs, with proportions of 14.39% and 0.72% respectively.

**Table 5:** Number and proportion of ADRs (n = 33,446).

ADR	N	Percentage	Cumulative percentage	ADR	N	Percentage	Cumulative percentage
Rash	5844	17.47	17.47	Hyperpyrexia	242	0.72	87.94
Pruritus	5212	15.58	33.06	Anaphylactic shock	242	0.72	88.67
Anaphylactoid reaction	4778	14.29	47.34	Back pain	229	0.68	89.35
Palpitation	2995	8.95	56.30	Rash maculo-papular	218	0.65	90.00
Dyspnoea	1641	4.91	61.20	Anaesthesia local	212	0.63	90.64
Nausea	1573	4.70	65.91	Rash erythematous	185	0.55	91.19
Dizziness	1385	4.14	70.05	Phlebitis	171	0.51	91.70
Rigors	1123	3.36	73.40	Coughing	160	0.48	92.18
Vomiting	1107	3.31	76.71	Asthenia	159	0.48	92.66
Flushing	1006	3.01	79.72	Cyanosis	158	0.47	93.13
Fever	664	1.99	81.71	Dermatitis	133	0.40	93.53
Headache	570	1.70	83.41	Tremor	133	0.40	93.92
Urticaria	482	1.44	84.85	Pallor	121	0.36	94.29
Pain	278	0.83	85.68	Diarrhoea	101	0.30	94.59
Sweating increased	270	0.81	86.49	Oedema	87	0.26	94.85
Abdominal pain	244	0.73	87.22	Malaise	79	0.24	95.08

### Outcome of ADRs

The vast majority of patients (99.24%) improved or recovered after treatment and intervention after the occurrence of ADRs (see Table 6).

**Table 6:** Number and proportion of reports on the outcome of ADRs (n = 25,416).

Outcome of ADRs	N	Percentage
Relieved	11,458	45.08
Cured	13,764	54.15
Not relieved	85	0.33
Left with sequelae	9	0.04
Death	9	0.04
Missing	91	0.36

Among the 9 deaths, 6 were males and 3 were females. The age distribution was relatively scattered, while there were slightly more elderly patients (> 70 years old, 4). The patients mainly suffered from respiratory, cardiovascular and cerebrovascular diseases as well as spinal diseases. The main ADRs were anaphylactic shock (5), dyspnoea (3) and anaphylactoid reaction (2) (see Table 7).

**Table 7:** Detailed information of the 9 deaths.

Case	Sex	Age	Suspected drug	Time^a^	Diseases	Dosage	ADR
1	Male	37	Dan hong injection	1	Open hand injury	40 ml	Palpitation, dyspnoea, opisthotonos, anaphylactic shock
2	Female	14	Xiyanping injection	2	Upper respiratory tract infection (URTI)	80 mg	Dyspnoea
3	Male	77	Safflower injection	0	Spinal disease	20 mg	Anaphylactic shock
4	Male	74	Shenmai injection	7	Chronic obstructive pulmonary disease (COPD), pulmonary heart disease (PHD), heart failure, ambulatory NYHA class IV	50 ml	Dyspnoea
5	Male	82	Shenmai injection	0	Coronary heart disease (CHD), cerebral infarction	40 ml	Anaphylactic shock
6	Female	73	Salvia injection	0	Essential hypertension	20 ml	Anaphylactic shock
7	Male	62	Acanthopanax senticosus injection	0	Heart failure, cerebral infarction, pulmonary heart disease (PHD)	400 mg	Anaphylactoid reaction
8	Female	51	Xiangdan injection	0	Spinal disease	20 ml	Anaphylactic shock
9	Male	50	Panax notoginseng saponins injection	0	Rheumatoid arthritis, bronchitis	400 mg	Anaphylactoid reaction

^a^Time means the occurrence time of the ADRs.

### Signal mining results

According to the calculation formulas and thresholds, DEC signals that do not meet the criteria were excluded. The ROR generated 19 signals, the PRR generated 19 signals, and the MHRA generated 123 signals. The three signal mining methods produced a total of 19 signals of the same DECs as shown in Table 8. The larger the ROR and PRR values, the stronger is the correlation between the drug and ADR.

**Table 8:** The signals of ADRs (3 methods).

TCM injection	ADR	ROR	95% CI lower limit	PRR	95% CI lower limit	χ^2^
Shuxuening injection	Phlebitis^a^	27.28	2.95	24.79	2.88	743.14
Shenmai injection	Back pain^a^	7.18	1.71	7.02	1.69	293.35
Ginkgolide injection	Phlebitis^a^	67.59	3.34	50.94	3.27	283.96
Kang-lai-te injection	Phlebitis	26.72	2.53	23.74	2.5	145.85
Qingkailing injection	Upper respiratory tract infection	61.06	2.64	60.81	2.63	96.11
Cinobufacini injection	Pain	28.91	2.38	23.54	2.36	85.7
Astragalus injection	Asthma	18.67	2.06	18.47	2.06	73.06
Salvianolate injection	Headache	6.66	1.37	6.08	1.33	62.27
Erigeron injection	Asthenia	5.09	1.03	5	1.03	32.02
Acanthopanax senticosus injection	Injection site pain	12.84	1.53	12.53	1.53	29.98
Aidi injection	Phlebitis^a^	5.78	1.04	5.65	1.03	25.02
Reduning injection	Injection site reaction^a^	7.25	1.09	7.21	1.09	21.3
Shuxuening injection	Dry mouth^a^	7.78	1.12	7.71	1.12	20.55
Java brucea fruit oil emulsion injection	Increased sweating	6.86	1.02	6.55	1.02	18.1
Xiyanping injection	Skin disorder	10.32	1.1	10.29	1.1	15.58
Xingnaojing injection	Allergic reaction	7.56	1	7.48	1	15.31
Salvia injection	Phlebitis superficial^a^	9.06	1	9.05	1	13.85
Shenmai injection	Renal pain^a^	25.09	1.03	25.06	1.03	13.33
Mailuoning injection	Vision abnormal^a^	8.9	1.01	8.81	1.01	12.78

^a^Off-label ADR.

## Discussion

Statistics showed that the reports of TCM injections reported by the spontaneous reporting system in the past 6 years were mostly from children and middle-aged and elderly patients. Wang also found that patients who suffered from ADRs caused by TCM injections were mainly over 50 years old. It was speculated that the occurrence of ADRs might be highly correlated with the patients’ own constitution, metabolism and the maturity and decline of organs^12^. Due to the limitations of data collection, it was impossible to know the usage frequency of TCM injections by age group.

In terms of the occurrence time of ADRs, most ADRs were found on the day of injection, and 96.41% of the ADRs occurred within one week. For patients injected with TCM injections, family members should be reminded to observe them closely on the day of injection and continue to observe them for a week so that most of the ADRs can be detected as early as possible and treated in time.

Combined with the severity of ADRs, the results showed that the severity distribution was not significantly different by sex. Some recent studies of children’s ADR reports from Chinese hospitals showed that the number of ADR reports of traditional Chinese medicine preparations (mostly TCM injections) is second only to antimicrobials. In addition, Li also indicated that TCM injections posed graver risks to children than adults, and in the paediatric population, TCM injections were significantly associated with anaphylactic shock^13^. It is important to note that although there are many reports of ADRs in children, the proportion of serious reports was the lowest. The possible reason was that doctors were more cautious in prescribing TCM injections when treating children. The rate of serious reports increased steadily with age, which may be associated with disease severity. The study suggested that the usage frequency of TCM injections in children and elderly patients should be reduced until the safety evaluation of TCM injections is improved.

The ADRs mainly involved skin and appendage damage, the body as a whole-general disorders and autonomic nervous system disorders, including rash, pruritus, anaphylactoid reaction and palpitation. Anaphylactic shock and anaphylactoid reaction were the most common serious ADRs of TCM injections, and posed a greater threat to patient safety or even death^8,14^.

Although the ADRs of most patients were improved or cured after treatment and intervention, there were still several cases of death and sequelae. Gender and age were not significantly different in death reports, and anaphylactoid reaction and anaphylactic shock accounted for the majority, consistent with previously reported results.

Under the background of TCM injections, some common ADRs of TCM injections such as nausea and vomiting do not generate signals in data mining, while certain ADRs of certain TCM injections may generate signals, which means that the ADR and TCM injection were probably related. Combined with drug instructions, off-label ADRs were found, including Shuxuening injection-phlebitis, Shuxuening injection-dry mouth, Shenmai injection-back pain, Shenmai injection-renal pain, Ginkgolide injection-phlebitis, Aidi injection-phlebitis, Reduning injection-injection site reaction, Salvia injection-phlebitis superficial and Mailuoning injection-abnormal vision.

Yang once analysed the 8-year ADR case reports of Shuxuening injection in the China SRS, indicating that phlebitis was a common symptom, and the warning signal of phlebitis was also obtained by Bayesian confidence propagation neural network, which was consistent with the ADR signal obtained in this study^15,16^. No studies have been found suggesting that dry mouth occurred with the use of Shuxuening injection. However, in view of the description of the reported data and the positive signal, more research could be done.

Wang conducted passive versus multicentre active surveillance, ADR case analysis, literature review and comprehensive safety research on Shenmai injection. Back pain was a common ADR and a warning signal of shenmai injection, which also supported the results of this study^17,18^. In addition, their research also suggested that there was no damage to renal function from Shenmai injection use at a dosage and a treatment course outside the recommended dosage and treatment course^18^. However, due to several cases with renal pain in the study who recovered after stopping the drug and the positive signal, the study implies that Shenmai injection may have an impact on renal pain, and more studies are needed.

There have been few studies on the ADRs of Ginkgolide injections. Liu analysed 120 related cases in a hospital for one year and found no ADRs^19^. Zhao found that 55 cases of Ginkgolide injection combined with atorvastatin calcium tablets had 1 case of dizziness and 1 case of nausea^20^. Perhaps due to the low incidence of ADRs and insufficient observed cases, the occurrence of ADRs cannot be observed. This study found a total of 8 cases of Ginkgolide injection-phlebitis and a positive signal, but the injections they used all came from the same manufacturer. The possibility of product quality as a cause of ADRs cannot be ruled out.

Phlebitis seemed to be an uncommon ADR of Aidi injection, but a few cases have been found in related studies^21^. This finding may be caused by insufficient solvent leading to poor dissolution and increased insoluble particles or the highly toxic cantharidin contained in the injection, which may cause phlebitis if the concentration is too high at the injection site^22,23^.

There were many reports of injection site reactions when Reduning injection was used, which supported the results of this study. Reduning injection contains three ingredients namely Artemisia annua, honeysuckle and gardenia. Artemisinin and its derivatives may cause injection site reactions, such as pain and swelling^24,25^. However, the specific mechanism is unclear.

No report of superficial phlebitis caused by Salvia injection was found, but there were some reports of phlebitis, which seemed to be related to rapid infusion^26,27^. The signal mining results had small ROR and PRR values, and the lower limit of the 95% CI was very close to 1, indicating a weak signal. Therefore, the association of superficial phlebitis or phlebitis with the drug could be suspected.

A number of studies and literature reviews on the ADRs of Mailuoning injection found several patients with conjunctival congestion or haemorrhage, which may indicate that eye damage was possible but rare^28–31^. A positive signal suggested that Mailuoning injection may be associated with vision abnormalities. In-depth research could be performed to uncover rare ADRs and their mechanisms of damage to the eyes.

In the instructions for TCM injections, ADRs are generally listed by items without detailed experimental or clinical data support, even though some of the instructions only record that ADRs are unknown. In the process of examining the instructions, it was found that the ADR items were approximately the same as the statistics for the number of ADRs of each TCM injection. Nevertheless, it was not completely clear whether the ADR was caused by the drug and whether there was a reasonable explanation of the mechanism. The data mining method can be used to obtain ADR signals of TCM injections with complex ingredients, which can be regarded as data support for ADRs in the instructions and is beneficial to the marketization and internationalization of TCM injections.

The statistical results and ADR signals obtained in this study are helpful in guiding the safe use of TCM injections in the clinic, and might be clues for ADR mechanism research, even providing advice for modifying drug labels based on the detection of off-label ADRs. In addition, this study has potential limitations. The effect estimated in the study is based on the data of a single province. Although the data are considerable, the external validity of the conclusion still needs to be improved. Due to the limitation of the selected signal mining method, the combination of drugs is not considered, and the conclusions may be biased.
